# Notes of an European Tour

**Published:** 1845-09-01

**Authors:** F. H. Hamilton

**Affiliations:** Professor of Surgery in Geneva Medical College


### BUFFALO MEDICAL JOURNAL

**Vol. 1.** SEPTEMBER 1, 1845. **No. 4.**

### Notes of an European Tour.---No. 4.

Communicated for the Buffalo Medical Journal, by F. H. HAMILTON, M. D., Professor of Surgery in Geneva Medical College.

GENEVA, SWITZERLAND.

DEAR M.—I can imagine your surprise in finding me thus suddenly transferred from the walls of Paris to the mountains of Switzerland; but I am *en route* for Italy, and perhaps for Egypt, and a few months later will render their heats intolerable to a Northerner. I shall spend a few weeks at Paris again on my return, and will then complete the sketch I have commenced of its hospitals, surgeons, &c.

I scarcely gave my friends at "Nos. 12 and 14" earlier notice of my intended change than I have given you, for as I was walking leisurely along rue St. Honore, I read over a stone gateway, "Messageries Generales :" — suddenly the mood was on me, and entering the large court around the interior of which are arranged the various offices from which diligences daily depart to almost every principal city on the continent; I secured immediately a seat for Geneva. It was now quite 2 o'clock P. M., and at 5 we passed out through the Barriere de la Gare, whose gates open towards Italy.

Before sunset we reached Fromenteau. At the same post at which we changed horses, Napoleon received intelligence of the capitulation of Paris to the allied troops. Passing ancient Essone, Pont-Thiery and Chailly, it was nearly midnight when we entered the gloomy forest of Fontainbleau, in the center of which stands the town of Fontainbleau, with its magnificent chateau. The moon was shining broad upon the vast and venerable pile, throwing into deep and striking contrast of light and shadow, its towering pavillions and deeply recessed archways : and as I remembered how many kings and queens with their lordly retinues had dwelt there, I listened long to hear if any sound of wassail came out from its many gilded halls, for it should be near the hour when princely revels commence, but all was silent as the grave; they sleep I know, for at St. Denis I have been down among their sepulchers,

> "In that deep vault,
> To whose foul mouth no healthsome air breaths in."

At Sens, an old Gallic town encircled by walls, we entered upon the beautiful valley of the Yonne, keeping upon its right bank through Villeneuve le Roy, and Villevillier, until we reached Joigny, the ancient

Joviniacum. At Auxerre, the capital of the department of Yonne, we again crossed the river and escaping from the valley, proceeded over a much more irregular country towards Avallon, passing the small towns of St. Bris, Vermanton and Lucy le Bois: Avallon was a fortified town before the subjugation of the Gauls by the Romans, and remains of a Roman road, I was told, might still be seen in its vicinity. From Avallon we contined to ascend gradually through Roubray, Maison Neuve, Vitteaux, La Chaleur and Pont de Pany to Dijon, the capitol of Cote d'Or. We were now near the sources of the Rhone, Seine and Moselle, and the air had become sensibly colder, and the soil less productive. Yet you will be surprised to learn that this is the country which produces the Burgundy wines, so celebrated in earlier days as to have given to the dukes of Burgundy the title of "Les Ducs Bons Vins." At Dijon I purchased for two francs a bottle of Chambertin, one of the red wines of Burgundy, and its rich, mellow, nutty flavor surpassed I thought any thing I had tasted in France. Dijon, famous the world over for its mustard; contains about 23000 inhabitants. Here is one of the secondary medical colleges, not authorized to grant degrees, with ten professors, and about thirty "eleves." M. Vallee, is Prof. of surgery, and M. Fleurot in the department of materia medica, is distinguished. Francis Chaussier was born at Dijon and became subsequently Prof. of chemistry and materia medica in this school, which was at that time greatly celebrated.—His contemporaries have called him "the most learned man in the world," being distinguished in almost every department of medicine surgery and natural science; he died at Paris in 1828.

Beyond Dijon we had the first view of the Jura mountains, in the distance; and after a ride of a few hours more, we reached Auxonne on the Saone, near which a considerable battle was fought between Napoleon and the allies; the conductor, a Frenchman, could not tell what battle, nor can I; if every battle-ground of Napoleon's was marked by a cross, the traveller here would need no guideboards, and if every soldier's grave had its marble slab, they might serve as mile stones for all Europe. Farther on we passed through the large town of Dole, whose strong fortifications were nearly demolished by Louis the XIV. While detained at the post I strolled through its beautiful promenade, and past its fine collegiate buildings, and was hurrying to take a peep at the ancient Roman road which once extended from the Rhone to the Rhine, when a shout from a "compagnon de voyage" with whom I had built a special intimacy, hurried me back to my place in the diligence. Leaving Dole we entered the fertile valley of the Glantine; highly cultivated fields extending along the whole route to Poligny, which is situated quite at the base of the mountains. After a brief delay at Poligny, where can always be found the delicious "vin de Mont Rachet," we commenced the ascent, taking the road built by Napoleon, smooth and perfectly secure to the very summit, and presenting at different points every variety of valley and mountain scenery. For many miles we had in view the beautiful and widely extended valley we had just left, covered with vinyards and wheat fields; farther up, the villages scattered along the mountains were picturesque and romantic in the extreme, being crowded occasionally into the smallest possible space within a mountain gorge, with only one street sufficiently wide for a carriage, and the rest mere goat paths; the houses built of stone even to the roofs, and sometimes partly or entirely excavated from the solid rock. Other hamlets hang upon the sides of the mountain, half way up their steep acclivity, and as seen from a distance appear wholly inaccessable to human footseps. Here and there also, a ruined chateau, or the remains of an ancient baronial castle crown the summit of a lofty and cragged peak, and for many miles may be kept in view; but in vain you strain your eyes to discover from within its walls signs of human life; no banner floats from its broken battlements, no mailed cavaliers in gay plumage wind from under its massive gateways; and it was well for us perhaps that a more gentle race, the wolf and vulture, now occupied these "robber nests," for in other days no unguarded troops like ours ever rode this way without paying full tribute to each lordly baron.

The peasantry of these mountains seem an industrious and hardy race. We began our ascent at about three in the morning, and as soon as the day began to dawn, the men were seen climbing the narrow footpaths along the mountains, some to their scanty vinyards or corn-fields, others driving a mixed flock of sheep and goats to browse among the rocks upon such stinted vegetation as their thin soil produced, the briar, wild gooseberry and alpine furze. And when the sun was well up, neat and ruddy women with wooden shoes, came clattering over the hills, bringing the simple morning repast, generally a few leeks or radishes, a loaf of black bread and a bottle of cheap wine, the product of their own small vintage.

Les Rousses is the French frontier town, but we were allowed to proceed without detention. Here we began our descent and at the first Swiss hamlet, La Vattey, I was reminded by the peculiar odour of the Inn that this was one of the manufacturing depots of that most offensive cheese denominated Gruyeries, which having once eaten at Paris and often smelt, I had no wish to become farther acquainted with. From La Vattey we took the road through St. Cergues. We had not descended far when emerging from a narrow mountain pass upon an open gallery, a view was presented suddenly and unexpectedly, such as I never before had seen and far exceeding my most lively conceptions. Before me lay an extended valley, "La Pays Vaud," covered with scattered villas, vinyards and lawns, and in its bosom slept lake Lemanus, with Geneva glittering in the noonday sun far down upon its outlet: while beyond, in the southeastern horizon arose the mighty Alps, forming a stupendous line of battlements whose icy turrets were lost and confounded in the white clouds which hung over them. How impossible is it to form any adequate conception of the vastness and majesty of the Alps until they have been seen: Surely in this first moment of rapturous gazing I was more than repaid for all my toil and dangers.

From the summit of the mountain to the plain, and thence along the banks of the lake, to Geneva, was but a few hours ride. At the gate we delivered our passports and crossing two bridges thrown across the "arrowy Rhone" and united by a small island, we were landed just at sunset at the Hotel de Geneve, dirty, hungry and weary, having rode 72 hours without sleep, and with only such eating as in France a country "auberge" affords, and what that is, you will know best when you travel this way.

At Geneva I have spent nearly a week, which has afforded me time to visit all places of special interest. My first business was to call upon Dr. H. C. Lombard, physician to the civil and military hospital of Geneva, whom I found exceedingly attentive, and to whom I am indebted for much valuable information. Dr. L. is not I think over 35 years of age, yet he has already greatly distinguished himself as a writer and practitioner. His medical education was acquired mostly in Paris, but he spent sufficient time in England to render him familiar with English practice, and to obtain such a knowledge of the language as to enable him to speak and write it handsomely. Although he had made his morning rounds, he kindly offered to accompany me to the hospital, a fine stone building forming a spacious court and situated in the upper part of the city. The first thing which arrested my special attention was the prevalence of goitre, with which not only the patients but the nurses almost without exception were more or less affected. To the inquiry whether it was not more common with women than men, Dr. L. replied that he did not think it was with unmarried women, but that in those districts where goitre most prevailed, its development was almost certain after child birth, and that even at Geneva, English and other foreign ladies were exceedingly apt to become affected with goitre immediately succeeding parturition. At Geneva and in its immediate vicinity, the proportion affected with this disease is not so large, but Dr. L. assured me, in the "Valais Canton" about two-thirds of the population were goitrous, the absence of the usual appendage being regarded as a deformity! In reference to its cause, a point so much in dispute, Dr. L. remarked that at Geneva, it was probably not true, as has been stated, that those who drank the lake water were less liable to the affection, than those who drank water only from certain springs in the city, and that in other parts of Switzerland I would find it prevailed mostly in deep valleys, and especially along those which extended North and South, and from which the direct rays of the sun were therefore mostly excluded. I also remarked to him what seemed to me to have some bearing upon the question, that the great mass of his hospital patients were scrofulous; almost every one, under whatever other malady they might be suffering, if the malady was chronic, had super-added also either enlargements of the glands, of the neck, or chronic opthalmia, or tumefaction of face or lips, or spinal distortion, or coxalgia, or enlargements of the knee, ankle, or of smaller joints. One, a girl about 17, I remember well, as presenting a most hideous picture, the very "tout ensemble" of scrofulous disfigurements; for in addition to many of the local affections already enumerated, her right eye was half protruded from its socket by an enormous irregular tumor, situated upon the antrum, discharging matter at several points, and the whole space between her chin and sternum was occupied by a large, nobby, ugly looking goitre.

The conclusion to which I have arrived then as to its cause, is that it depends upon the same causes, only slightly modified, which usually develope scrofula, an opinion which I shall hold with the right of change, until I have myself visited the goitrous districts, as I propose soon to do.

"First of all," says Dr. L., "the patient, if we would cure him, must be removed from the valley to the mountain, and then" adds Dr. L., "I consider iodine as much a specific, as quinine is for intermittent fever, and quite as certain, provided the remedies are applied early." He exhibits iodine both internally and externally, having become of late somewhat cautious to avoid iodization, an event which is indicated by a general and rapid marasmus, hectic, and often speedy death. In the only instance in which Dr. L. had seen the thyroid gland removed, the patient died of tetanus ; Dr. Bizot, the surgeon, however, remarked that he had removed safely encysted goitre, but would never attempt the removal of a simple thyroid hypertrophy. He also stated that he had found the "huile de foie de morue"—cod liver oil, a most excellent substitute for iodine in certain cases, and that he had also extended its use beneficially to cases of simple scrofula ; the muriate of gold also he relied much upon in scrofula. The observations of these men I regard of unusual value, from the acknowledged accuracy and honesty of the gentlemen and their unequalled familiarity with the diseases in question. In the surgical wards of Dr. Bizot I saw nothing strikingly peculiar ; the straight splint, in its simplest form, is here generally employed, and indeed as the Swiss surgeons are generally educated at Paris, their surgery differs but little from that of the Parisian hospitals.

The second day I spent in a stroll through the city, composed of a mixed population, speaking French, German and Italian ; the French language and customs are however greatly predominant. The city is divided into the upper and lower town, in the former of which reside the ancient Genevese aristocracy, in the latter the poorer class of citizens, merchants, artizans, &c. That part of the lower town however, which borders immediately upon the lake and is situated upon the banks of the Rhone, boasts of many large and elegant buildings. Most of the streets are narrow and crooked, and between the old and new town so steep as to render their ascent often exceedingly difficult.

You have heard it remarked doubtless that travellers had found a resemblance between Geneva in New-York and Geneva in Switzerland, and I ought to tell you wherein the resemblance *lies*. If the outlet of Seneca lake was a mighty "rushing" stream, whose waters were shaded with the richest tints of blue and green, instead of a small sluggish and yellow creek ; if Geneva *chez nous*, stood quite upon its outlet, instead of a mile above and upon the terraced banks of the lake alone ; if the streets were narrow, crooked, dark, damp and paved with solid blocks of stone, instead of straight and open every where to the broadest light of day, except where the maple and acacia lend their delightful shade ; if its private dwellings were lofty brick edifices, ranging upon the streets, entered by heavy gateways, and the lower windows secured with bars of massive iron, instead of small, neat, white cottages, with modern doors and green window blinds, each house retiring from the road to make room for a beautiful *parterre de gazon* in front ; if its churches were huge masses of ancient masonry, and its inns Astor houses, with princely accommodations, instead of light and graceful specimens of American architecture, and tidy country-houses, with reasonable yankee comforts ; if the village of five thousand inhabitants were a compact walled city of 30,000 ; and more, if instead of being surrounded by an extensive and gently undulating plain, it lay deeply embosomed between mountains "whose vast walls have pinnacled in clouds their snowy scalps," then would one of the most lovely villages of our new republic,

> "Which stands amid the seven fair lakes that lie,
> Like mirrors neath the summer sky,"

resemble the capital of the free and ancient Allobrogi. Each is beautiful but unique, and to say they are alike, is to rob them both.

I have visited the church of "St. Pierre," in which Calvin preached, a noble and venerable building; I entered the pulpit from which, near three hundred years since, he thundered his denunciations against the impurities of Papacy; I descended and sat where his auditors had sat, upon the same old curiously carved seats. I was also anxious to visit his tomb, and went from the church to the public cemetery, about a mile outside the southern gate of the city. Over the entrance I read "Hereux ceux qui meurent au Seigneur ! Ils se reposent de leur traveaux et leurs œvres les suivent." How beautiful and impressive!

The gate was open, and I asked a grey-headed sexton to point me the tomb of Calvin: and I was surprised that he persisted in saying he "Je ne sais pas." Finding me, however, exceedingly importunate, he conducted me to a spot near the southeast wall of the cemetry, and pointing to a rude bench near a small tree, he said "it is generally believed he is buried near that bench; he is not in the old cemetry, but somewhere in this. I believe it must be about *there*, but do not know positively, nor does any one know ; by his own request no mark was ever made to indicate the place of his burial." I gathered a few rank weeds and a couple of large slimy snails which were crawling upon the (in my faith,) consecrated ground, the only mementoes I could find, and which I have carefully prepared, and shall keep for my good Mother.

Returning, a space in the rear of the theatre, not far from the ramparts, was indicated to me as that in which in 1794, during the reign of terror, the scaffold and guillotine were erected, and many of the most respectable citizens of Geneva were executed. It is now a botanic garden, made fertile by human blood.

It reminds me also to say that here in 1553, Michael Servetus, a medical and surgical writer of great celebrity, was burnt at the stake. The circumstances of his death are thus related. Servetus had become a reformer, but in his opinions had far outstrode the doctrines of Calvin, who was then preaching at Geneva, while Servetus was publishing anonymously his sentiments at Vienna. Calvin, who was professedly his friend, and whom Servetus had made a confident, betrayed him to the authorities of Vienna, and he was immediately arrested and imprisoned, but escaping, and still ignorant of the true author of his exposure, he fled to Geneva. Calvin here again procured his arrest, and a charge of blasphemy and heresy being brought against him, he was convicted and burnt ! In the work which led to his death, he had spoken of the passage of the blood through the lungs, a fact in physiology before unknown, and which may be considered a near approach to the discovery of the circulation of the blood.

Geneva, I may say, in passing, has been distinguished as the birthplace of many eminent medical men, with whose names the medical scholar must be familiar—of Mayerne in the year 1573, who was successively physician to Henry IV of France, to James 1st, and Charles 1st and 2d of England—of Bonet the pathologist in 1620—of Le Clerc, author of "Histoire de la Medecine," &c. and of Manget, chief physician to Frederick 3d, King of Prussia, in 1652. Geneva also, was the birthplace of Jean Jacques Rousseau, Neckar, Saussure, and Sismondi. Am I not already treading upon classic ground!

Yesterday the air was so cheering that I determined to walk to the residence of Merle d' Aubigne, author of the recent great work upon the reformation. He lives at "Eaux Vivant" about two miles from Geneva, and after a delightful walk I found his villa, called "La Campagne de Merle d' Aubigue", surrounded by a small park extending in the rear to the shore of the lake. M. d' Aubigne received me with an agreeable ease and courtesy, and I spent an hour with him very pleasantly. He is, I should think, about 45, tall and well formed, with dark complexion, black hair and eyes, and a rather meditative, but exceedingly pleasant expression of face. He speaks English well, and we talked chiefly of Zwingle, and Calvin, and Papacy, which he declared was on the increase in Switzerland —by emigration however, rather than by conversion. He seemed gratified that his writings had been republished in America, for he had always felt a deep sympathy with all Americans, with several of whom he had become acquainted, and he inquired after them with apparent interest. When I left he grasped my hand with affectionate warmth and commended me to the care of the Great Protector.

I have been to Ferney once the residence of a man to whom Merle d' Aubigne is doubtless an exact antipode. The villa of Voltaire is about five miles from Geneva, near the foot of the Jura mountains. It stands almost as he left it, an old gruff looking chateau, built upon a slight eminence and surrounded by a handsome, but now somewhat neglected park. The "salle de reception" and "chambre a coucher" remain, the attendant assured me, precisely as they were during the occupancy of the "great literary demigod". I am pleased that this has been permitted, since I am enabled to read in the furniture of these two apartments more than volumes of biography could teach. All things here are in such strange and inexplicable contrasts, and groupings as were ever blending in their owners mind, and such as have defied either his cotemporaries or posterity to unite upon his character. The hired panegyrist of an Emperor, and bold subverter of Empires—the satyrist of pomp and ostentation, and the very soul of pride and conceit in himself—a patriot and self exiled—a philosopher and wit—poet and historian—moralist and libertine—an atheist, yet so devout that Spurzheim believed excess of devotion had made him such! Against the dingy walls of these two apartments hang portraits of Frederick the Great, and Washington—Catharine 2d Empress of Russia, and his pretty Laundress—his virtuous niece, and his rouged *chere amie*, Madame de Chatelet—Milton and Lekain—Newton and Corneille—a pet chimney sweep and a nude Venus —Benjamin Franklin in the costume of an American Indian—a picture of his Henriade designed by himself, in which he who wrote "si Dieu n' existait pas, il foudrait, l'inventer" is once consistent and has apotheosized *himself!* Underneath a small engraving of Franklin is written, (composed by Voltaire,)

> "Homme du noveau monde et de l'humanite,
> Ce sage amiable et vrai, las guide et les eclaire;
> Comme un autre Mentor, il cache a l' oeil vulgare
> Sous les traits d'un mortel, une divinite."

The bed, chairs, toilet table, washstand, &c., are such as might become the cell of an anchorite.

Without, and near one angle of the chateau is a small stone chapel, erected by Voltaire to the God whose existence he denied. The stone over the door bearing the inscription "Deo erexit Voltaire" was torn away by the reformers. Beside it stands a marble monument to his memory; his heart is in the Pantheon at Paris. From the chapel I strolled through the gardens in the rear of the house and along the coppiced arbor where he used to walk; it is nearly 70 years ago that as a feeble, tottering, emaciated, irritable old man, the ancient gardener now lingering also upon the verge of life, remembers to have followed him through these avenues.

It was approaching sunset, when, returning from Ferney, and about three miles from Geneva, it was my remarkably good fortune to see Mont Blanc unveiled to its very summit. There are not on an average more than sixty days in the year in which this mountain is not wrapt in clouds. "Look! look! said my conductor, "Mont Blanc! Mont Blanc!"—and for quite fifteen minutes I saw this hoary headed Titan, while the sun was gradually sinking behind the Jura mountains; and for yet fifteen minutes more after he had set to us, could it be seen changing slowly from a pale white, to a dull, deathlike, leaden hue, such as I have seen come gradually over the face of a dying man.

